# Generation of Biologically Active Multi-Sialylated Recombinant Human EPOFc in Plants

**DOI:** 10.1371/journal.pone.0054836

**Published:** 2013-01-25

**Authors:** Alexandra Castilho, Laura Neumann, Pia Gattinger, Richard Strasser, Karola Vorauer-Uhl, Thomas Sterovsky, Friedrich Altmann, Herta Steinkellner

**Affiliations:** 1 Department of Applied Genetics and Cell Biology, University of Natural Resources and Life Sciences, Vienna, Austria; 2 Department of Chemistry, University of Natural Resources and Life Sciences, Vienna, Austria; 3 Department of Biotechnology, University of Natural Resources and Life Sciences, Vienna, Austria; 4 Polymun Scientific GmbH, Klosterneuburg, Austria; TGen, United States of America

## Abstract

Hyperglycosylated proteins are more stable, show increased serum half-life and less sensitivity to proteolysis compared to non-sialylated forms. This applies particularly to recombinant human erythropoietin (rhEPO). Recent progress in *N*-glycoengineering of non-mammalian expression hosts resulted in *in vivo* protein sialylation at great homogeneity. However the synthesis of multi-sialylated *N-*glycans is so far restricted to mammalian cells. Here we used a plant based expression system to accomplish multi-antennary protein sialylation. A human erythropoietin fusion protein (EPOFc) was transiently expressed in *Nicotiana benthamiana* ΔXTFT, a glycosylation mutant that lacks plant specific N-glycan residues. cDNA of the hormone was co-delivered into plants with the necessary genes for (i) branching (ii) β1,4-galactosylation as well as for the (iii) synthesis, transport and transfer of sialic acid. This resulted in the production of recombinant EPOFc carrying bi- tri- and tetra-sialylated complex *N-*glycans. The formation of this highly complex oligosaccharide structure required the coordinated expression of 11 human proteins acting in different subcellular compartments at different stages of the glycosylation pathway. *In vitro* receptor binding assays demonstrate the generation of biologically active molecules. We demonstrate the *in planta* synthesis of one of the most complex mammalian glycoforms pointing to an outstanding high degree of tolerance to changes in the glycosylation pathway in plants.

## Introduction

Recombinant human erythropoietin (rhEPO) was the first hematopoietic growth factor approved to treat anemia associated with kidney failure, cancer and other pathological conditions [1]. Mature EPO is a 30 kDa glycoprotein with 166 amino acids carrying three *N-*linked (Asn-24, -38 and -83) and one O-linked (Ser-126) carbohydrate chains which account for 40% of the total molecular weight [2], [3]. Glycosylation has a profound effect in maintaining the overall stability and *in vivo* hematopoietic activity of hEPO [4]–[6]. Several studies report that terminal sialic acid increases the circulatory half- life of rhEPO, moreover a positive correlation between the *in vivo* biological activity and the ratio of tetra- to bi-antennary sialylated oligosaccharides was shown [7], [8]. Due to the complexity of the glycosylation pattern, therapeutic rhEPO is exclusively produced in mammalian cell cultures, mainly in Chinese hamster ovary (CHO) [9]–[11]. Many efforts have been made to improve the sialylation content of the hormone [12]–[14]. Indeed, hyper-sialylated rhEPOs with prolonged half-life and subsequent enhanced drug efficacy were produced [6]. Another strategy to improve drug efficacy of rhEPO is its fusion to stabilizing peptides/proteins. The application of immunoglobulin Fc-fusions to therapeutic proteins has become very popular since the Fc fragment can extend the conjugated protein serum half-life by being recycled via the neonatal Fc receptor (FcRn). EPOFc fusions have been successfully explored in this direction [15].

The limited production capacity and expensive mammalian cell based production facilities make the recombinant hormone very costly. A viable alternative for the large-scale and low cost production of biopharmaceuticals is the use of plants [16], [17]. Recent progress in expression levels, production speed and up-scaling, have placed this expression system into an encouraging position. Another important feature of using plants as production platform is their ability to carry out human-like complex N-glycosylation. Due to their comparable small repertoire of glycosylation reactions, plants carry out complex *N*-glycosylation with remarkable homogeneity, which makes them especially amenable for *N*-glycoengineering. Indeed, over the past years many research groups have concentrated their efforts on modulating plant *N*-glycosylation to enable the production of recombinant proteins with human-like structures (Review [18], [19] One of the most impressive results is the introduction of the mammalian biosynthetic pathway for *in planta* protein sialylation [20].

Previous attempts to produce rhEPO in plants resulted in the generation of a recombinant hormone that shows *in vitro* activity [21]–[23]. However, plant-derived rEPO was not active *in vivo* most probably due the lack of sialylation [24]. Regrettably, most of these studies did not consider the glycosylation status of the recombinant hormone. rhEPO and rhEPOFc produced in glycoengineered moss and *N. benthamiana* carried mainly human type complex GlcNAc_2_Man_3_GlcNAc_2_ (GnGn) structures, lacking plant specific xylose and fucose [25], [26]. Moreover the production of rhEPOFc with tetra-antennary bisected complex *N-*glycans was achieved upon overexpression of mammalian *N-*acetylglucosaminyltransferases (GnTIII, GnTIV and GnTV) [26], [27]. Overall, the results demonstrate the feasibility of plants to generate active rhEPOFc with a targeted N-glycosylation profile, however plant derived (multi-) sialylated rhEPOFc remains elusive.

In this investigation we set out to express in plants rhEPOFc carrying tri- and tetra-sialylated N-glycans. Agrobacterium containing rhEPOFc cDNA was delivered to *N. benthamiana* ΔXTFT mutants (lacking the plant specific *N*-glycan residues β1,2-xylose and core α1,3-fucose) together with the mammalian genes required for *in planta* protein sialylation (i.e. 6 genes, [20]). *N*-glycosylation profiling of the recombinant protein using LC-ESI-MS exhibited the synthesis of mainly complex bi-antennary sialylated *N*-glycans, i.e. NeuAc_2_Gal_2_GlcNAc_2_Man_3_GlcNAc_2_ (NaNa). Transient co-expression of rhEPOFc with mammalian genes necessary for the branching and sialylation of *N*-glycans (in total 11 genes) resulted in the synthesis of rhEPOFc decorated with tri- and tetra-sialylated oligosaccharides. All glycoforms exhibit biological activities comparable to the CHO derived rhEPOFc, as determined by cell-based receptor binding assays.

## Materials and Methods

### Vectors for single gene expression

MagnICON pro-vector system was used for the expression of rhEPOFc chimeric proteins as described before [26]. For modulation of rhEPOFc N-glycosylation profile we used the previously described binary vectors each carrying a single gene necessary to produced multi-antennary *N-*glycans (^FUT11^GnTIV and ^FUT11^GnTV, [26]) and to assemble *in planta* the metabolic pathway for protein sialylation (GNE, NANS, CMAS, CST, ^ST^GalT and ST, [20]).

### Binary vectors for multiple gene expression

**Figure 1:**
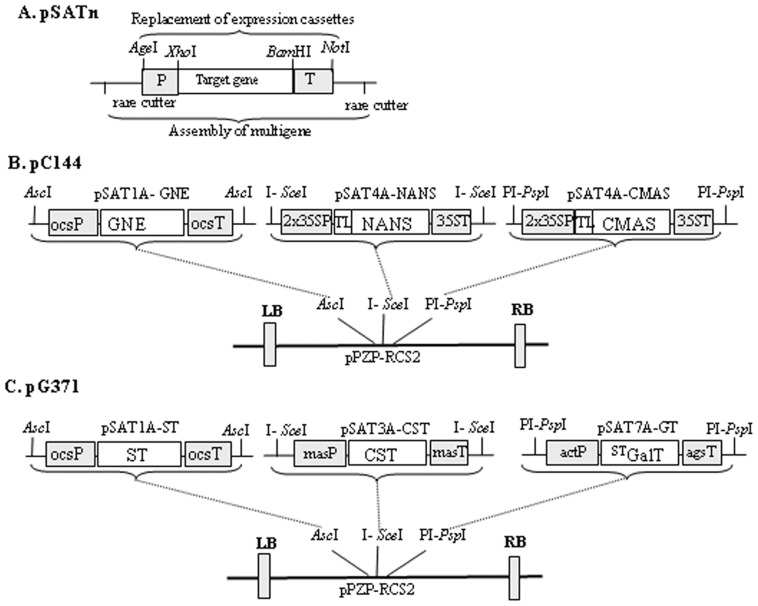
Schematic representation of the multi-gene vectors used in this investigation. A. Structural features of the pSAT series of vectors (pSATn) suitable for the expression of target genes under the control of various constitutive promoters and terminators (pSAT1A, pSAT3A, pSAT4A and pSAT7A). Expression cassettes are interchangeable within pSATn as *Age*I-*Not*I fragments. Rare-cutting enzymes flanking each pSAT vector are used to transfer the expression cassettes into the expression vector pPZP-RCS2. B. Outline of the cloning strategy to assemble the mammalian genes necessary for the synthesis of sialic acid, pC144. The GNE, NANS and CMAS [20] open reading frames were subcloned into pSAT auxiliary vectors and were then sequentially assembled in pPZP-RCS2 using specific rare-cutting enzymes. C. Outline of the cloning strategy to assemble the mammalian genes acting in the Golgi apparatus for *in planta* protein sialylation, pG371. ^ST^GalT, CST and ST [20] genes were put under control of different promoters and terminators in pSAT vectors. These were then sequentially assembled into pPZP-RCS2 vector using appropriate rare-cutting enzymes. 35SP: cauliflower mosaic virus (CaMV) 35S promoter; TL: translational enhancer 5′-UTR from tobacco etch; 35ST: CaMV 35S terminator; OcsP: octopine synthase promoter; OcsT: octopine synthase terminator; actP: actin promoter; agsT: agropin synthase terminator; masP: manopine synthase promoter masT: manopine synthase terminator; GNE: mouse UDP-*N-*acetylglucosamine-2-epimerase/*N-*acetylmannosamine kinase; NANS: Homo sapiens *N-*acetylneuraminic acid phosphate synthase; CMAS: Homo sapiens CMP-*N-*acetylneuraminic acid synthase; ^ST^GalT: β1,4-galactosyltransfease fused to the cytoplasmic tail, transmembrane domain and stem region of the rat α2,6-sialyltransferase; CST: Mouse CMP-sialic acid transporter; ST: rat α2,6-sialyltransferase; LB: left border; RB: right border.

We combined six expression cassettes in two different binary plasmids: one for the expression of the genes necessary for the synthesis of sugar-activated sialic acid, CMP-Neu5Ac (GNE, NANS and CMAS) and another for the expression of genes necessary for synthesis of the acceptor substrate (β1,4-galactosylation), Golgi transport and transfer of sialic acid (CST, ^ST^GalT and ST). To this intent we used the versatile pSAT family that allows target genes to be cloned under a large choice of promoters and terminators that are easily interchangeable (Fig 1A, [17]). cDNA from each gene were amplified from the correspondent binary vector described in Castilho et al [20]. Appropriate rare-cutting enzymes flanking the expression cassettes in each pSAT vector were used to assemble several cassettes into plant transformation RCS2-based vectors carrying the same rare-cutting enzymes [17]. The pSAT auxiliary vectors and the pPZP-RCS2 binary vectors were purchased from University of Michigan, USA.

### Construction of vector for the expression of GNE, NANS and CMAS

The cDNA of GNE was amplified with primers GNE R1/F1 digested with *Xho*I/*BgL*II and cloned into pSAT1A digested *Xho*I/*Bam*HI (pSAT1A-GNE). NANS and CMAS cDNAs were amplified with primers NANS F1/R1 and CMAS F1/R1 respectively, digested with *Xho*I/*Bam*HI and cloned into pSAT4A (pSAT4A-NANS and pSAT4A-CMAS). The expression cassette of pSAT4A-CMAS was transferred into the *Age*I-*Not*I sites of the pSAT6A. To obtain the construct for simultaneous expression of the three proteins the expression cassette of pSAT1A-GNE was removed by *Asc*I digestion and cloned into the *Asc*I site of pPZP-RCS2, the expression cassette from pSAT4A-NANS was removed by I-*Sce*I digestion and cloned into the I-*Sce*I site of pPZP-RCS2 and finally the CMAS expression cassette was inserted into the site PI-*Psp*I of pPZP-RCS2 (pC144, Figure 1B)

### Construction of vector for the expression of CST, ^ST^GalT and ST

The cDNA from CMP-Neu5Ac transporter was amplified from the correspondent binary vector with the primer pair CST F1/R1, digested with *Xho*I/*Bam*HI and cloned into pSAT3A digested the same way (pSAT3A-CST). cDNA from the modified β1,4-galactosyltransferase (^ST^GalT [28]) was amplified with ST F1/STGalT R1 primers digested and cloned into the *Xho*I-*Bam*HI sites of pSAT7A (SAT7A-GT). Lastly, the cDNA from the rat α2,6-sialyltranferase was amplified with the primer pair STF1/R1 digested with *Xho*I/*Bam*HI and cloned into pSAT1A (pSAT1A-ST). Upon restriction with appropriate rare-cutting enzymes, the pSAT1A-ST, pSAT3A-CST and pSAT7A-GT were assembled into pPZP-RCS2 vector as *Asc*I, I-*Sce*I and PI-*Psp*I fragments, respectively (pG371, Figure 1C).

All binary vectors were transformed into the *Agrobacterium tumefaciens* strain UIA 143 and magnICON constructs were transformed into strain GV3101 pMP90. All primers used in this investigation are listed in Table S1.

### Plant material and transient protein expression

*Nicotiana benthamiana* ΔXTFT plants [29] were grown in a growth chamber at 22°C with a 16 h light/8 h dark photoperiod.

Transient expression of rhEPOFc was done in four-to-five-week old plants by agroinfiltration. The magnICON 3′- vector containing cDNA was co-infiltrated with the corresponding 5`-vector carrying the signal peptide for secretion in combination with the binary vector for the expression of the recombinase [30]. For modulation of the *N*-glycosylation profiles, binary vectors containing the cDNA of the different mammalian genes were co-infiltrated with the magnICON viral-based vectors. Agrobacteria carrying the magnICON constructs were infiltrated using optical density (OD_600_) 0.1 and 0.05 for agrobacteria carrying binary constructs.

### Protein purification, N-glycan analysis and peptide mapping

rhEPOFc was purified from agroinfiltrated leaves (200–300 mg) with rProteinA Sepharose™Fast Flow (GE Healthcare) as described previously [26]. For glycopeptide analysis, purified rhEPOFc were resolved by SDS-PAGE and bands corresponding to 55 kDa were cut out, S-alkylated and double-digested with trypsin and endoproteinase Glu-C. This double digestion allows site-specific analysis of all four *N*-glycosylation sites (GPs): EPO GP1: E/A^22^ENITTGCAE^31^; EPO GP2: E/H^32^CSLNENITVPDTK^45^, EPO GP3: R/G^77^QALLVNSSQPWEPLQHLVDK^97^ and Fc glycopeptide: R/EEQYNSTYR. Subsequently samples were analysed by liquid-chromatography electrospray ionization-mass spectrometry, LC-ESI-MS [31], [32]. Briefly, a BioBasic C18 column (150×0.32 mm, 5 µm; Thermo Scientific) was eluted with 0.3% formic acid buffered to pH 3.0 with ammonia as the aqueous solvent and a gradient from 10% to 55% acetonitrile developed over 40 min of 1.5 µL/min. The glycoforms of a given peptide co-eluted due to the use of buffered eluent [32]. The elution zone of each peak was summed and the spectra were deconvoluted using MaxEnt3 (Waters Micromass). Peak heights were taken as indicators of the molar ratios of glycoforms, which was recently shown to give meaningful results for Fc-glycopeptides [31].

The 30 kDa protein band corresponding to free Fc was analysed by LC ESI MS/MS for peptide mapping in order to identify the N-terminus. The data was analyzed using the X! Tandem open source software to match tandem mass spectra with the EPO-Fc protein sequence. The N-terminal peptide was identified by the GPM (Global Protein Machine) search engine.

### Immunoblot Analysis

Five micrograms of total soluble protein and Protein A purified rhEPOFc were subjected to 12% SDS-PAGE under reducing conditions and blotted onto Hybond Enhanced Chemiluminescence nitrocellulose membranes (GE Healthcare). The blots were blocked in 1xPBS containing 0.1% (v/v) Tween 20 and 3% (w/v) BSA for 1 h and the protein bands were analysed by immunoblotting using either anti-hEPO (1∶3000 dilution MAB2871, R&D Systems, Minneapolis, MN), anti-human IgG (1∶5000 dilution anti-Fc, W4031 Promega, Mannheim, Germany) or anti-Lewis-A (1∶40 dilution JIM84, kindly provided by Paul Knox, University of Leeds, UK) antibodies.

### rhEPOFc quantification and in vitro assay

The expression level of plant-derived rhEPOFc was measured in total soluble proteins using the Quantikine IVD ELISA for human EPO (DEPOO, R&D Systems) according to manufacturer's instructions. The biological activity of protein A purified rhEPOFc was measured in a UT-7 cell based proliferation assay. Briefly, the UT-7 cell line [33] was maintained in RPMI 1640 (Biochrome AG) supplemented with 10% fetal calf serum (PAN Biotech.), 4 mM L-glutamine and 5 ng/mL EPO. The cells were washed with EPO free culture medium and incubated for 4 h at 37°C and 7% CO_2_. In a 96-well culture plate increasing amounts of CHO-derived rhEPOFc (0.009–60 ng/ml) and plant-derived rhEPOFc (ranging from 0.003–20 ng/mL) were added to 100 µL of medium containing about 10^4^ cells. After 4 days at 37°C and 7% CO_2_, 10 µL of a MTT (Thiazolyl Blue Tetrazolium Bromide; Sigma) solution (5 mg/mL) were supplied to each well and the plate was incubated for 4 h as before. Finally, 100 µL of 10% SDS (in 0.01 M HCl) were added to each well and mixed thoroughly at 37°C before reading absorbance at 570 nm (reference wavelength 690 nm). The experiments were performed in 5 replicates and the results were evaluated using MS Excel Solver. The half maximal effective UT-7 cell proliferation dose (ED_50_) was used to compare the activities of plant- and CHO-derived rhEPOFc.

## Results

### Transient expression of EPOFc in N. benthamiana ΔXTFT

**Figure 2:**
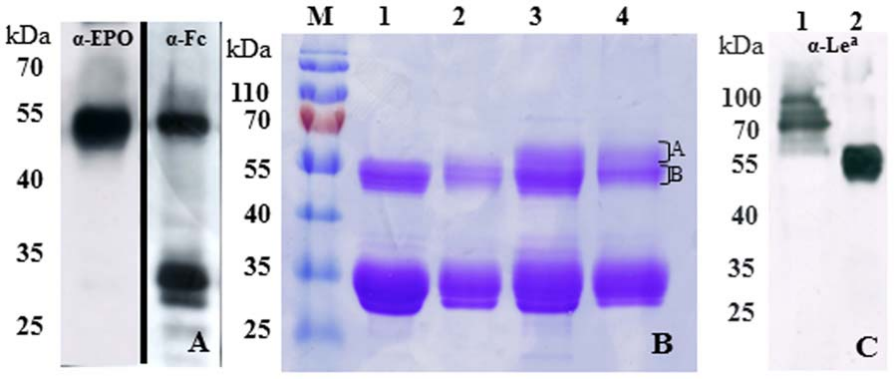
Expression of rhEPOFc in *N. benthamiana*. A. Western blot analysis of total soluble proteins extracted from *N. benthamiana* expressing rhEPOFc. 55 kDa protein reacts to both anti-EPO (α-EPO) and anti-Fc (α-Fc) antibodies while the ∼30 kDa band reacts only with α-Fc antibodies. B. Protein A purified rhEPOFc fractionated by SDS PAGE and stained with Coomassie-brilliant blue R-250. lane 1: rhEPOFc expressed in *N. benthamiana* mutants lacking plant specific β1,2-xylose and α1,3-fucose (rhEPOFc_ΔXTFT_); lane 2: rhEPOFc co-expressed with mammalian genes for protein sialylation (GNE, NANS, CMAS, CST, ^ST^GalT and ST) (rhEPOFc_Sia_,); lane 3: rhEPOFc co-expressed with mammalian genes necessary for sialylation and synthesis of tri-antennary *N-*glycans GnTIV or GnTV, (rhEPO_TriSia_,); lane 4: rhEPOFc co-expressed with mammalian genes for sialylation and synthesis of tetra-antennary *N-*glycans, GnTIV and GnTV (rhEPO_TetraSia_). A and B represent distinct protein fractions from the 55 kDa band of rhEpoFc_TriSia_ and rhEPO_TetraSia_, used for N-glycan analysis; the ∼30 kDa band represent free Fc. C. Western blot analysis of total soluble proteins (5 µg TSP) extracted from *N. benthamiana* ΔXTFT mutants (control; lane 1) and of purified rhEPOFc_ΔXTFT_ (lane 2) using antibodies against Lewis-A epitopes (JIM 84). Several proteins in TSP and the 55 kDa protein band corresponding to intact rhEPOFc reacted to JIM 84 revealing the presence of *N-*glycans with Lewis-a epitopes. (M) protein marker.

We used *N. benthamiana* ΔXTFT, a glycosylation mutant that synthesizes complex *N-*glycans devoid of plant specific β1,2-xylose and core α1,3-fucose, as expression platform [29]. In previous studies we have shown the versatility of these plants for the modulation of plant *N*-glycosylation towards mammalian-like structures (recently reviewed [19]). Using the potent viral-based expression system magnICON, [30] appropriate agrobacteria carrying hEPOFc cDNA were delivered to ΔXTFT leaves. 4–5 days post infiltration (dpi) expression was monitored by Western blotting. Antibodies against EPO and Fc enabled the detection of a 55 kDa band which corresponds to the expected size of the fusion protein and an additional 30 kDa band that reacted only with anti Fc antibodies (Figure 2A). The expression level of the intact protein was up to 9 mg/kg leaves, corresponding to 0.2% of total soluble protein (Table 1). rhEPO was purified via protein A-based chromatography and separated by SDS PAGE. Coomassie staining revealed the presence of two bands as already detected by immunoblotting (Figure 2B). Peptide mapping and MS analyses demonstrated that the 55 kDa band corresponds to the intact rhEPOFc, while the 30 kDa band refers to free Fc (data not shown). Similar observations of rhEPOFc fragmentation have been reported in earlier studies in transgenic chickens [34] and in plants [26], [27]. In our attempts to enhance the expression of full-length rhEPOFc, different fusion constructs were generated. These included plant codon-optimization of the hEPO fragment using the GeneArt® Gene Synthesis and GeneOptimizer® process (www.lifetechnologies.com, GenBank accession No. KC329647), amino acid variations in the hinge region of Fc, and exchange of the hinge-Fc fragment from IgG1 for the IgGD hinge and the IgG4-Fc regions [35]. Another concern is the post translational elimination of the arginyl (Arg^166^) amino acid residue. Analysis of the C-terminus of CHO-rhEPO and human EPO purified from the urine demonstrates that the Arg^166^ predicted to be at the C- terminus is missing. This is presumably due to the enzymatic activity of endogenous carboxypeptidases [36]. Since plants contain several types of carboxypeptidases the trimming of Arg^166^ and the consequent loss of tags fused to the C-terminus cannot be excluded. To possibly prevent this eventual cleavage we generated a hEPOFc fusion lacking this amino acid. Unfortunately none of the strategies led to improved expression of full length EPOFc (data not shown). Moreover, the identification of the N-terminus on the free Fc fraction by LC ESI MS/MS was not clear and the results showed that the ∼30 kDa band consist of a mixture of Fc fragment fused to varying sizes of EPO sequence. It was therefore not possible to identify an exact cleavage site between the hEPO and the Fc.

**Table 1:** Expression of rhEPOFc in *N. benthamiana*.

rhEPOFc	mg/kg	% TSP
ΔXTFT	9.13	0.18
Sia	6.14	0.12
TriSia	9.12	0.18
TetraSia	9.11	0.18

Concentration of transiently expressed rhEPOFc was determined using a commercially available immunoassay. For each sample the concentration is given in mg/kg of fresh leaf. The percentage of the total soluble protein (TSP) was also calculated. rhEPOFc was expressed in *N. benthamiana* ΔXTFT mutants (ΔXTFT); co-expressed in ΔXTFT with mammalian genes for protein sialylation (Sia); co-expressed in ΔXTFT with mammalian genes for synthesis of tri-antennary sialylated *N-*glycans (TriaSia) and co-expressed in ΔXTFT with mammalian genes for synthesis of tetra-sialylated *N-*glycans (TetraSia)

**Figure 3:**
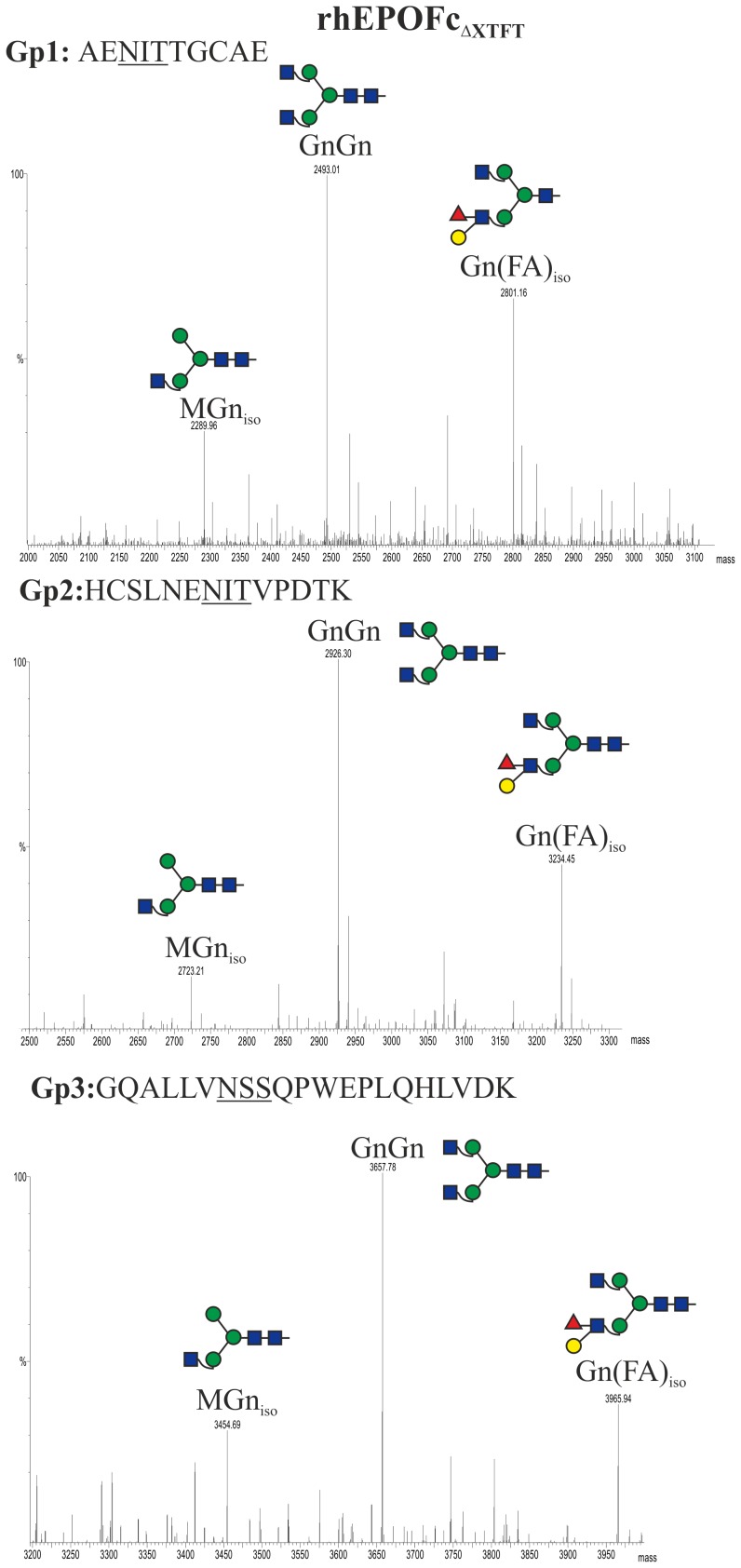
Generation of GnGn structures in rhEPOFc. Mass spectra of trypsin and endoproteinase Glu-C double-digested rhEPOFc expressed in *N. benthamiana* ΔXTFT (rhEPOFc_ΔXTFT_; Figure 2B, lane 1). Glycosylation patterns of rhEPO glycopeptide 1 (Gp1): E/A^22^ENITTGCAE^31^; glycopeptide 2 (Gp2): E/H^32^CSLNENITVPDTK^45^ and glycopeptide 3 (Gp3): R/G^77^QALLVNSSQPWEPLQHLVDK^97^ are shown. The corresponding *N-*glycosylation profile of the Fc glycopeptide (R/EEQYNSTYR) is shown in Figure S1. Peak labels were made according to the ProGlycAn system (www.proglycan.com). Illustrations display *N*-glycans on assigned peaks, for interpretation of other assigned glycoforms see Figure S5.

LC-ESI-MS analysis was performed to determine the *N*-glycosylation profile of purified rhEPOFc expressed in ΔXTFT (rhEPOFc_ΔXTFT_, Figure 2B, lane 1). MS data revealed that all three *N*-glycosylation sites of rhEPO carry a similar glycosylation pattern (Figure 3), with a major glycoform, GnGn. In addition significant amounts of structures compatible to Gn(FA)_iso_ were present, a carbohydrate formation already detected previously on plant derived rhEPO and rhEPOFc [25], [26]. The presence of the terminal trisaccharide consisting of α1,4-fucose and β1,3-galactose linked to *N-*acetylglucosamine also known as Lewis-a epitope can be detected by immunoreaction to the monoclonal antibody, JIM84 [23], [37]. Total soluble proteins (TSP) and protein A purified rhEPOFc_ΔXTFT_ analysed by Western blotting showed that the 55 kDa band corresponding to the intact rhEPOFc reacts with anti-Lewis-a antibodies, while the free Fc 30 kDa band does not (Figure 2C). In fact, the glycosylation profile of Fc exhibits exclusively GnGn structures (Figure S1).

### Multiple gene expression vectors

In previous studies we have shown that *in planta* sialylation can be accomplished by co-infiltration of 6 agrobacteria cultures into a plant leaf (each carries a binary vector with a mammalian glycosylation gene) [20]. To achieve this, all recombinant proteins (including the target protein, which is also co-delivered) must work in the same cell in a highly coordinated fashion. However, the infection of a single cell via agro-infiltration is a random procedure, thus the delivery of single constructs might lead to inefficiencies. To facilitate the simultaneous delivery of all cDNAs to the same cell, two multi gene vectors were generated, each carrying three mammalian glycosylation genes. The pSAT-family vectors allow target genes to be cloned under a large choice of promoters and terminators and the expression cassettes are easily interchangeable (Figure 1A [17]). The six different cDNAs were initially cloned into pSAT vector and subsequently groups of three expression cassettes were assembled in two binary vectors: (i) pC144, carries the genes necessary for the synthesis of nucleotide sugar activated sialic acid, CMP-Neu5Ac (GNE, NANS and CMAS, Figure 1B); (ii) pG371, carries the genes necessary for the synthesis of the β1,4-galactosylated acceptor substrate, Golgi transport and transfer of sialic acid (CST, ^ST^Gal and ST, Figure 1C). For detailed description of the vectors see Experimental Procedures.

### Generation of bi-sialylated N-glycans on rhEPOFc

**Figure 4:**
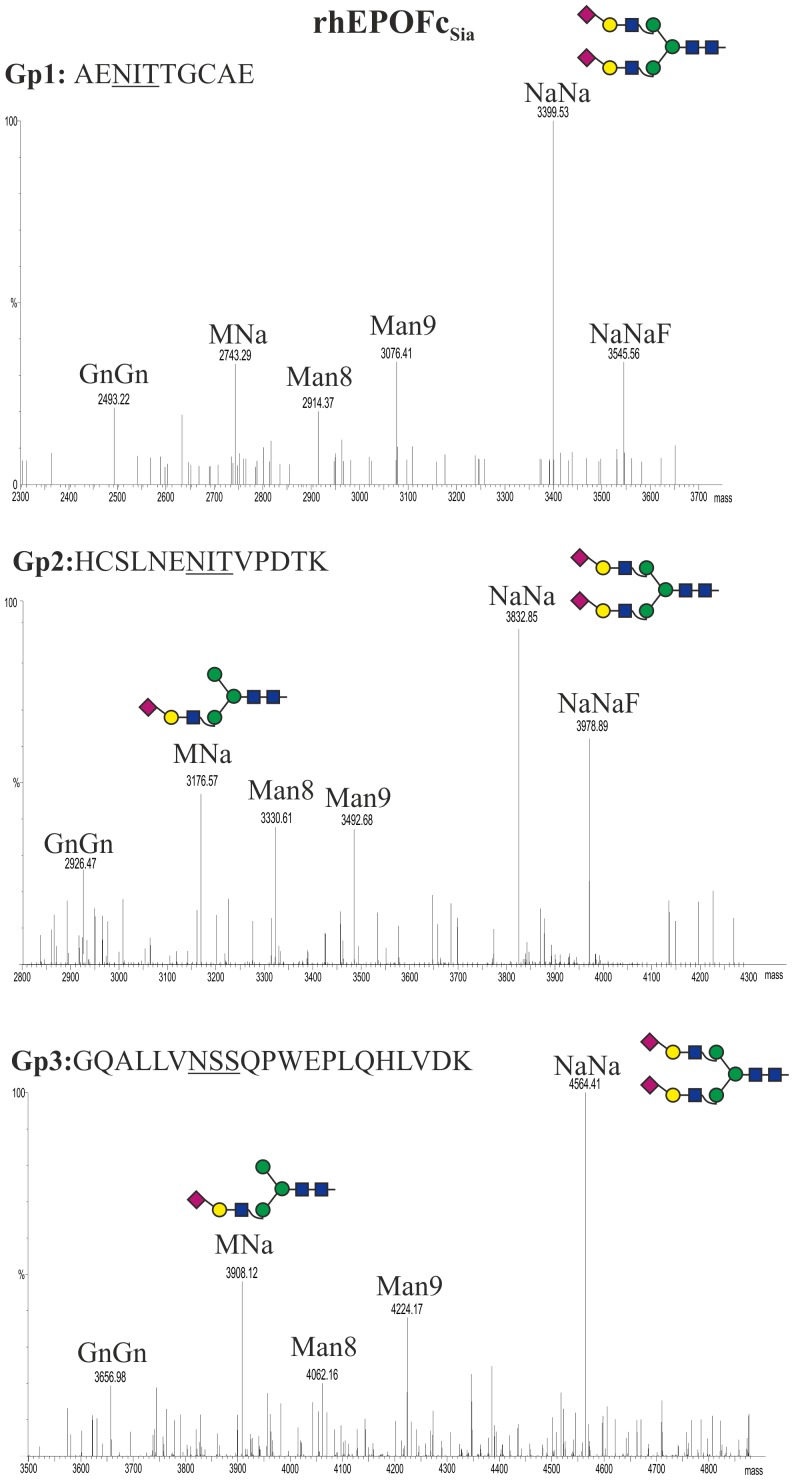
Generation of bi-sialylated structures in rhEPOFc. Mass spectra of trypsin and endoproteinase Glu-C double-digested rhEPOFc co-expressed in *N. benthamiana* ΔXTFT with mammalian genes for protein sialylation (GNE, NANS, CMAS, CST, ^ST^GalT and ST) (rhEPOFc_Sia_; Figure 2B, lane 2). Glycosylation patterns of rhEPO Gp1: E/A^22^ENITTGCAE^31^; Gp2: E/H^32^CSLNENITVPDTK^45^ and Gp3: R/G^77^QALLVNSSQPWEPLQHLVDK^97^ are shown. *N-*glycosylation profile of the Fc glycopeptide is shown in Figure S1. Peak labels were made according to the ProGlycAn system (www.proglycan.com). Illustrations display *N*-glycans on assigned peaks, for interpretation of other assigned glycoforms see Figure S5.

In order to elongate the GnGn glycoforms present on rhEPOFc_ΔXTFT_ with β1,4-galactose and α2,6-linked sialic acid, the hormone was co-expressed with the multi gene vectors, pC144 and pG371, allowing a total of 9 genes to be simultaneously delivered to ΔXTFT. Site specific *N*-glycosylation-profiling of the purified recombinant hormone (rhEPOFc_Sia_, Figure 2B, lane 2) showed that all *N-*glycosylation sites on the rhEPO are similarly occupied and were efficiently modulated (Figure 4). MS analysis of the 55 kDa band revealed that about 90% of complex *N*-glycans was sialylated (Table 2). Notably, we observed a dominant *N-*linked glycoform, i.e. bi-antennary sialylated structures (NaNa), which accounts for more than 60% of all complex structures. In addition fucosylated (NaNaF) and incompletely sialylated (MNa) glycoforms were detected and about 10–15% of rhEPOFc_Sia_ carried oligomannosidic structures (not included in Table 2). Surprisingly, no Lewis-a structures were detected. In contrast, the *N-*glycan profile of Fc exhibited a largely heterogeneous glycosylation profile, including GnGn, mono and bi-galactosylated structures (GnA, AA), incompletely processed structures (MNa) and oligomannosidic glycoforms (Figure S1). Notably, the procedure worked in a similar way when single binary vectors were used [20].

**Table 2:** Relative abundance of different complex glycoforms detected in rhEPOFc. (oligomannosidic structures that are present in all samples are not included).

	EpoFc_Sia_			EpoFc_TriSia_			EpoFc_TetraSia_		
Glycoform (%)	Gp1	Gp2	Gp3	Gp1	Gp2	Gp3	Gp1	Gp2	Gp3
GnGn	9.1	10.1	10.9	4.8	3.7	6.6	5.6	4.4	3.4
**MNa_iso_**	**12.1**	**17.6**	**22.5**	-	-	-	12.3	-	11.2
**NaNa**	**62**	**49.7**	**66.6**	**4**	**12.4**	**15.3**	**13.5**	**29.4**	**26.9**
**NaNaF**	**16.8**	**22.6**	-	-	-	-		-	-
[GnGn]Gn				18.8	10.1	10.4	10.7	2	2.1
[GnGn]GnF				-	2.2	-	-	-	-
[AGn]Gn				-	2.1	-	-	-	-
[AGn]GnF				-	1.4	-	-	-	-
**[NaNa]Na**				**72.4**	**68.1**	**67.7**	**56.3**	**39.6**	**21.5**
[GnGn][GnGn]							1.6	7.7	10.4
[GnGn][GnA]_iso_							-	5.3	9.7
[GnA][GnA]_iso_							-	1.2	1.3
**[NaNa][NaNa]**							-	**10.4**	**13.5**
**∑ Sialylation**	**90.9**	**89.9**	**89.1**	**76.4**	**81.5**	**83**	**82.1**	**79.4**	**73.1**

Relative abundance of complex *N*-glycans determined by LC-ESI-MS. **rhEPOFc_Sia_**: rhEPOFc co-expressed with mammalian genes for protein sialylation; **rhEPOFc_TriaSia_**: rhEPOFc co-expressed with mammalian genes for synthesis of tri-antennary sialylated *N-*glycans; **rhEPOFc_TetraSia_:** rhEPOFc co-expressed with mammalian genes for synthesis of tetra-sialylated *N-*glycans. ΔXTFT was used as expression host. Values are in percentages. Quantifications were done for complex *N-*glycans (oligomannosidic structures were not included in calculations). Gp1: glycopeptide 1; Gp2: Glycopeptide 2; Gp3: Glycopeptide 3.

### Generation of multi-sialylated N-glycans on rhEPOFc

**Figure 5:**
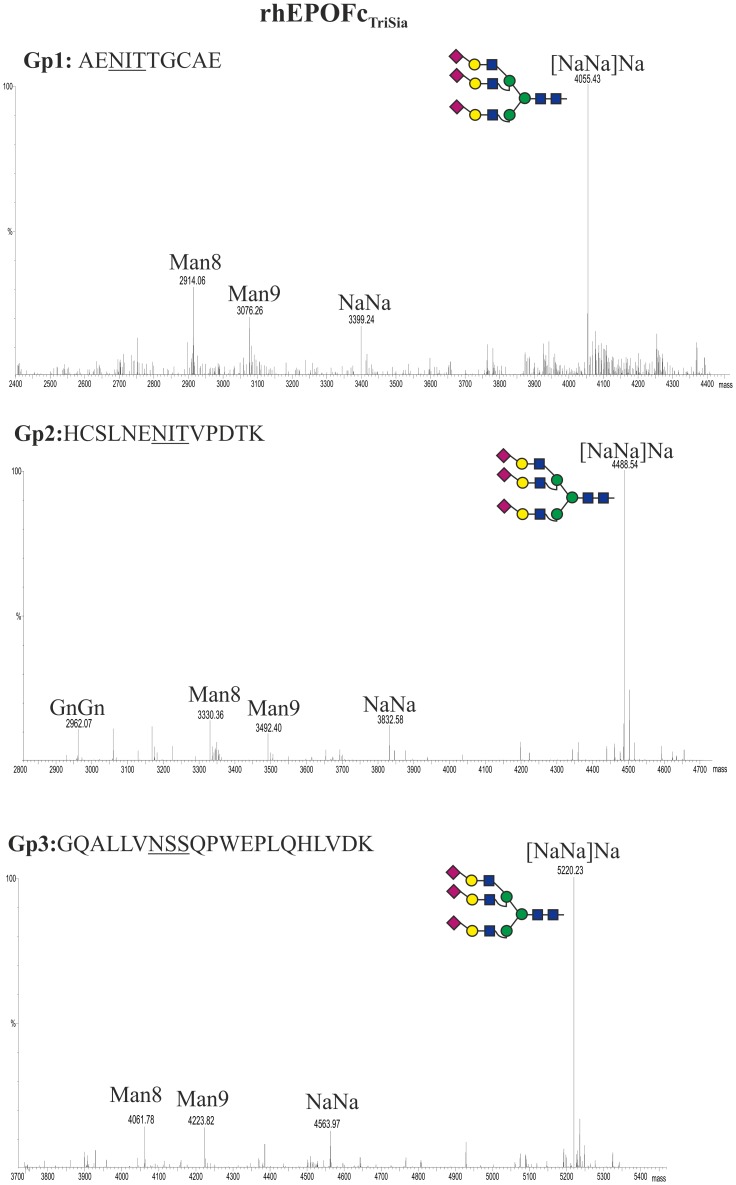
Generation of tri-sialylated structures in rhEPOFc. Mass spectra of trypsin and endoproteinase Glu-C double-digested rhEPOFc co-expressed in *N. benthamiana* ΔXTFT with mammalian genes for synthesis of tri-antennary sialylated *N-*glycans (rhEPO_TriSia_). The analysis was performed on rhEPOFc_TriSia_ present on fraction A of the 55kDa band (Figure 2B, lane 3). Glycosylation patterns of rhEPO Gp1: E/A^22^ENITTGCAE^31^; Gp2: E/H^32^CSLNENITVPDTK^45^ and Gp3: R/G^77^QALLVNSSQPWEPLQHLVDK^97^ are shown. *N-*glycosylation profile of the Fc glycopeptide is shown in Figure S1. Glycosylation profile of rhEPOFc present on fraction B of the 55 kDa band is shown in Figure S2. Peak labels were made according to the ProGlycAn system (www.proglycan.com). Illustrations display *N*-glycans on assigned peaks, for interpretation of other assigned glycoforms see Figure S5.

The generation of plant derived rhEPOFc carrying branched (tri- and tetra-antennary) *N*-glycans has been reported previously [26], [27]. This was achieved by the co-expression of rhEPOFc with mammalian *N-*acetylglucosaminyltransferases IV and V targeted to medial Golgi compartment (^FUT11^GnTIV or ^FUT11^GnTV; [26]). Here we set out to generate multi-antennary sialylated rhEPOFc. To approach this issue, we first co-expressed rhEPOFc with pC144 and pG371 in combination with either ^FUT11^GnTIV or ^FUT11^GnTV. SDS-PAGE analysis of purified rhEPOFc (rhEPOFc_TriSia_, Figure 2B lane 3) showed that the 55 kDa band corresponding to the fusion protein appears as a “smeary” band compared to rhEPOFc_ΔXTFT_ or rhEPOFc_Sia_ (Fig 2B, lanes 1 and 2, respectively). The relative occurrence of the different complex glycoforms present in rhEPOFc_TriSia_ is displayed in Table 2. In total about 80% of all glycans were sialylated, with the dominant *N*-glycan, being tri-sialylated oligosaccharide. Low amounts of tri-antennary non-sialylated structures are also detected and oligomannosidic structures account for ca. 10–12% of the total *N-*glycans. In addition the “smeary” 55 kDa band was separated into two fractions (A and B, Figure 2B) and they were individually analysed. The *N*-glycosylation profile of fraction A (which corresponds to a size slightly larger than 55 kDa) exhibits almost exclusively tri-antennary sialylated carbohydrates in all three glycosites ([NaNa]Na) (Figure 5). In contrast, fraction B (which corresponds to the lower part of the 55 kDa band) was decorated mainly with tri-antennary non-sialylated *N*-glycans with or without galactosylation ([GnGn]Gn, [AGn]Gn), accompanied by oligomannosidic *N-*glycans (Figure S2).

Finally rhEPOFc was co-expressed with pC144 and pG371 in combination with both ^FUT11^GnTIV and ^FUT11^GnTV. This procedure encompasses a coordinated action of eleven heterologous proteins. The purified product (rhEPOFc_TetraSia_) exhibited on Coommassie stained SDS-PAGE a “smeary” 55 kDa band as observed for rhEPOFc_TriSia_.

**Figure 6:**
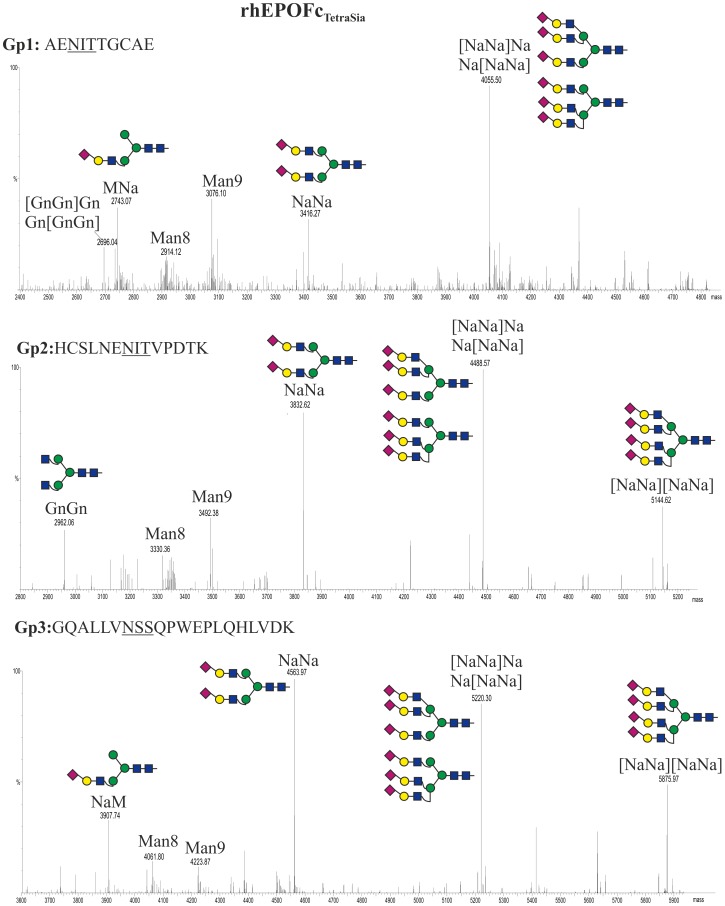
Generation of tetra-sialylated structures in rhEPOFc. Mass spectra of trypsin and endoproteinase Glu-C double-digested rhEPOFc co-expressed in *N. benthamiana* ΔXTFT with mammalian genes for synthesis of tetra-sialylated *N-*glycans (rhEPO_TetraSia_). The analysis was performed on rhEPOFc_TetraSia_ present on fraction A of the 55kDa band (Figure 2B, lane 4). Glycosylation patterns of rhEPO Gp1: E/A^22^ENITTGCAE^31^; Gp2: E/H^32^CSLNENITVPDTK^45^ and Gp3: R/G^77^QALLVNSSQPWEPLQHLVDK^97^ are shown. *N-*glycosylation profile of the Fc glycopeptide is shown in Figure S1. Glycosylation profile of rhEPOFc present on fraction B of the 55kDa band is shown in Figure S3. Peak labels were made according to the ProGlycAn system (www.proglycan.com Illustrations display *N*-glycans on assigned peaks, for interpretation of other assigned glycoforms see Figure S5.

LC-ESI-MS analysis revealed that rhEPOFc_TetraSia_ glycopeptides carried about 80% sialylated structures including tri- and tetra-sialylation (Table 2). Tri-antennary sialylated structures were the major glycoform in all three rhEPO glycosites (up to 56%). While GP 2 and 3 carried about 10-13% tetra-sialylated structures, surprisingly, this complex carbohydrate was not present on GP 1. Moreover ∼15% of rhEPOFc_TetraSia_ are decorated with oligomannosidic structures (not included in Table 2). As before, *N*-glycosylation analysis was individually performed on the two fractions A and B. (Figure 2B, lane 4). The main glycoform of rhEPOFc in fraction A is tri-sialylated with significant amounts of bi- and tetra-sialylated *N*-glycans (NaNa and [NaNa][NaNa]) on glycopeptide 2 (Gp2, Asn-38) and Gp3 (Asn-83) (Figure 6). Interestingly, on Gp1 (Asn-24) a single dominant peak corresponding to tri-sialylated structures is detected as well as smaller fractions of bi-sialylated glycans (NaNa) but no tetra-sialylated N-glycans were detected (Figure 6). Fraction B exhibited a variety of non-sialylated branched *N-*glycans some carrying one or two galactose residues ([GnGn][GnGn], Gn[GnGn]_iso_, GnGn, [GnGn][GnA] and [GnA][GnA]_iso_. Consistently with fraction A, Gp1 carries only GnGn and tri-antennary *N*-glycans (Figure S3).

Analysis of Fc glycosylation in rhEPoFc_TriSia_ and rhEPOFc_TetraSia_ shows a largely heterogenous *N*-glycosylation profile with a mixture of GnGn and oligomannosidic glycoforms, but also minor amounts of tri-antennary, galactosylated and sialylated structures (Figure S1). Notably expression levels of all glycoforms were in the same range (Table 1) indicating that co-infiltration of human glycosylation enzymes did not alter expression level of the recombinant fusion protein.

### In vitro activity of different EPOFc glycoforms

Finally the biological activity of the plant-derived rhEPOFc variants was analysed using an erythropoietin-dependent human leukemia cell line, UT-7. Proliferation of the UT-7 cells is induced by the presence of EPO. The proliferation of UT-7 cells was measured and half maximal effective dose (ED_50_) values were compared. All plant-derived rhEPOFc glycoforms had similar ED_50_ values ranging 0.26–0.54 ng/mL. Comparably a slightly reduced receptor binding was obtained for the CHO derived counterpart (ED_50_ 1.7 ng/mL) (Table 3). This might be due to different downstream procedures of plant and CHO derived recombinant hormones, e. g. CHO derived rhEPOFc but not the plant derived counterparts was subjected to a virus inactivation test.

**Table 3:** *in vitro* activity of CHO- and plant-derived rhEPOFC.

rhEPOFc	ED_50_ (ng/mL)
CHO	1.7
ΔXTFT	0.45
Sia	0.54
TriSia	0.25
TetraSia	0.26

*In vitro* activity assay of plant- and CHO- derived rhEPOFc. Half maximal effective doses (ED_50_) are displayed. rhEPOFc was expressed in CHO cells (CHO); in *N. benthamiana* ΔXTFT mutants (ΔXTFT); co-expressed in ΔXTFT with mammalian genes for protein sialylation (Sia); co-expressed in ΔXTFT with mammalian genes for synthesis of tri-antennary sialylated *N-*glycans (TriaSia) and co-expressed in ΔXTFT with mammalian genes for synthesis of tetra-sialylated *N-*glycans (TetraSia).

## Discussion

With the recognition of the N-glycan nature of the AB0 blood group types, glycoconjugates were accepted to elicit specific reactions [38]. Since then numerous studies have highlighted the impact of this important posttranslational modification on the function of proteins. A well-known example is rhEPO, one of the leading biopharmaceutical products. The human EPO is a highly glycosylated molecule with three *N*- and one *O*-linked glycans. The relevance of *O*-glycosylation for the biological activity of EPO is unclear, and implications for a role in secretion are not conclusive [39]. On the contrary the biological implications of *N-*glycosylation are well characterized [4]–[6]. In the course of enhancing drug efficacy in anemia treatment, increased *in vivo* half-life *via* enhanced terminal sialylation was achieved. Moreover, fusing the hormone to an IgG-Fc domain resulted in a significant extension of the serum half-life of the recombinant hormone [40]. Here we report the transient expression of rhEPOFc in plants. Using the *N. benthamiana* ΔXTFT in combination with the magnICON based expression system, we achieved expression levels of rhEPOFc of up to 9 mg/kg leaves, which accounts for 0.2% of TSP. This is a relatively modest expression level in comparison to amounts reported for other recombinant proteins with the magnICON systems [30], [41], however they are in agreement with rhEPOFc expressed previously in plants [27]. Low expression could result from the fact that a large portion of the recombinantly expressed protein (about 30–50 times) refers to Fc lacking the hEPO fragment. We designed different rhEPOFc chimeras to address this issue. However neither manipulation on the Fc-hinge region nor the presence/absence of the EPO Arginine^166^ residue had a significant influence on expression of the full length fusion protein. The generation of free Fc has been already reported previously upon expression of hEPOFc in chicken [34], however it is not present when produced in mammalian cells [9], [42]. The reason for this phenomenon has not been investigated in detail, although degradation of the fusion protein by plant proteases is a plausible explanation. Several studies refer to the proteolytic degradation of heterologous proteins in plants [43] and the outcome indicated that this occurs preferentially in the apoplast [44]. Importantly human proteins like EPOFc have not evolved in the context of plant proteases and thereby they represent novel targets. The apoplastic fluid of *N. benthamiana* is enriched of acidic proteases, e.g. the presence of papain-like cystein family was reported [45]. Papain is a non-specific protease that cleaves monoclonal antibodies preferentially in the *N-*terminal side of the hinge region and was effectively used to separate the rhEPO from the Fc fragment during *N*-glycan profiling of rhEPOFc produced in CHO cells [9]. Proteolysis is a major issue of recombinant proteins affecting the product yield, not only in plants but also in other expression systems. Different strategies are being considered to avoid or minimize proteolysis of heterologous proteins expressed in plants [46]. And the outcome hopefully will allow enhanced expression of full length rhEPOFc in plants.

Here we report the generation of rhEPOFc glyco-variants which largely resembles that of the CHO derived counterparts (Figure S4). Expression of rhEPOFc in ΔXTFT mutants results in the formation of almost exclusively GnGn structures on all glycosylation sites. Interestingly, although present in total soluble proteins extracted from ΔXTFT mutants and in some recombinantly expressed proteins [47], no truncated paucimannosidic structures, i.e. MM, were detected. Co-expression of hEPOFc with the mammalian genes involved in protein sialylation permitted the production of a hormone largely decorated with bi-antennary sialylated complex *N-*glycans. These structures are one of the major glycoforms of EPO present human serum [48], however accounts only for about 15% on the CHO derived counterpart [49]. Moreover, we report the synthesis of plant-derived rhEPOFc carrying multi-antennary sialylated *N-*glycans, the major structures of mammalian cell derived therapeutic rhEPO. Co-expression of rhEPOFc with the genes necessary for *N-*glycan branching and sialylation resulted in a mixture of neutral and charged oligosaccharides. In total approximately 10–16 glycoforms, with different relative amounts, are distributed by the four glycopeptides, similar to the observation for CHO-derived rhEPOFc [9]. In summary, rhEPOFc carried 80–90% sialylated structures upon co-expression with the sialylation pathway. Bi- and tri-sialylation were the major glycoform in recombinant products rhEPOFc_Sia_ and rhEPOFc_TriSia_, respectively. In contrast to mammalian cell derived rhEPO, tetra-sialylation is inefficiently synthesized in plants and rhEPOFc_TetraSia_ carries only about 10–14% of this highly complex carbohydrate formation. Beside complex *N-*glycans, plant derived rhEPOFc carries about 10–15% oligomannosidic structures. Notably, and as for mammalian cells, there are significant differences on the *N*-glycan profile of rhEPO and of Fc domain. While efficient modulation towards mammalian-like structures was observed on all rhEPO glycosites, Fc glycosylation exhibited unusual structures. A homogeneous glycosylation profile (namely GnGn oligosaccharides) was obtained for Fc produced in ΔXTFT, all further modification steps (branching, sialylation) led to the synthesis of a largely heterogeneous glycosylation profile with unusual incompletely processed structures. In particular, sialylation was modest. The unusual Fc glycan-modulation was already observed previously for the synthesis of tetra-antennary *N-*glycans in rhEPOFc [26]. The reason for this different performance in glyco-modulation of rhEPO and Fc is currently not understood. One explanation could be different accessibility of the *N*-glycosylation sites, EPO glycosites are considered very exposed while the Fc-glycosites are buried within the protein backbone [50] and as a consequence they have restricted accessibility to *N*-glycan processing enzymes.

Multi-sialylation of rhEPOFc requires the coordinated expression of 11 exogenous genes in a single cell. To reduce the number of agrobacteria cultures and to facilitate the simultaneous delivery of glycosylation genes into the same cell, two multi gene vectors carrying the six genes for *in planta* sialylation were constructed (pC144 and pG371). With a future intention of using these vectors to stably introduce the sialic acid pathway into plants, different plant selection markers have been placed to the vector backbones. Also a combination of several promoter and terminator sequences were used to reduce the risk of transgene silencing when attempting to express a series of genes stably from a single plasmid. Here, by transient expression, we demonstrate that these multi-gene vectors efficiently sialylate their target protein making them valuable tools for plant transformation.

Transgenic *N. benthamiana* plants stable expressing mammalian glycosyltransferases can be extremely useful for the production of recombinant proteins with a highly homogenous human-like glycosylation profile as recently demonstrated [27], [28].

Importantly all plant-derived rhEPOFc glycoforms are biologically active as seen in receptor binding assays. These results are a good starting point for follow up advanced structure-function studies, with the aim to determine the most suitable glycoforms. These will be the focus of future experiments.

With the generation of multi-sialylated glycans we display *in vivo* engineering of one of the most complex human *N*-glycan structures *in planta* and thereby demonstrate the enormous plasticity of plants to tolerate modifications on their protein *N-*glycosylation. The results presented here together with other achievements in plant *N-*glycoengineering (reviewed by [19]) provide the know-how for the generation of recombinant proteins with targeted *N-*glycosylation profiles. This allows advanced protein-carbohydrate structure-function studies to better understand the impact of *N*-glycans and to develop next generation drugs, where patients would benefit from optimally glycosylated drugs.

## Supporting Information

Figure S1 ***N-*** **glycosylation profile observed in the Fc glycopeptide (R/EEQYNSTYR) of rhEPOFc_ΔXTFT_: rhEPOFc expressed in** ***N. benthamiana*** **ΔXTFT mutants; rhEPOFc_Sia_: rhEPOFc co-expressed in ΔXTFT with mammalian genes for protein sialylation; rhEPO_TriSia_: rhEPOFc co-expressed in ΔXTFT with mammalian genes for synthesis of tri-antennary sialylated** ***N-*** **glycans; rhEPO_TetraSia_: rhEPOFc co-expressed in ΔXTFT with mammalian genes for synthesis of tetra-sialylated** ***N-*** **glycans.** For interpretation of glycoforms present in assigned peaks see Figure S5.
(TIF)

Figure S2 ***N*** **-glycosylation profile of rhEPOFc_TriSia_ present in fraction B of the 55kDa band (** **Figure 2B** **, lane 3).** Glycosylation patterns of rhEPO Gp1: E/A^22^ENITTGCAE^31^; Gp2: E/H^32^CSLNENITVPDTK^45^ and Gp3: R/G^77^QALLVNSSQPWEPLQHLVDK^97^ are shown. Peak labels were made according to the ProGlycAn system (www.proglycan.com). For interpretation of glycoforms present in assigned peaks see Figure S5.
(TIF)

Figure S3 ***N*** **-glycosylation profile of rhEPOFc_TetraSia_ present in fraction B of the 55kDa band (** **Figure 2B** **, lane 4).** Glycosylation patterns of rhEPO Gp1: E/A^22^ENITTGCAE^31^; Gp2: E/H^32^CSLNENITVPDTK^45^ and Gp3: R/G^77^QALLVNSSQPWEPLQHLVDK^97^ are shown. Peak labels were made according to the ProGlycAn system (www.proglycan.com). For interpretation of glycoforms present in assigned peaks see Figure S5.
(TIF)

Figure S4 ***N*** **-glycosylation profile of rhEPOFc expressed in CHO cells.** Glycosylation patterns of rhEPO Gp1: E/A^22^ENITTGCAE^31^; Gp2: E/H^32^CSLNENITVPDTK^45^; Gp3: R/G^77^QALLVNSSQPWEPLQHLVDK^97^and the Fc glycopeptide (R/EEQYNSTYR) are shown. Peak labels were made according to the ProGlycAn system (www.proglycan.com). For interpretation of glycoforms present in assigned peaks see Figure S5.
(TIF)

Figure S5 **Illustration of** ***N*** **-glycan structures on transiently expressed rhEPOFc.** Oligomannosidic structures (Man5–Man9), complex *N*-glycans typical of plant-derived proteins (GnGnXF) and glycans carrying Lewis-a epitopes (Gn(FA)_iso_) are also illustrated. Schematic representations are based on the nomenclature proposed by the consortium for Functional Glycomics.
(TIF)

Table S1 **List of primers as cited in Material and Methods.**
(DOCX)
