# Supplementary figures and images for: Generation of Biologically Active Multi-Sialylated Recombinant Human EPOFc in Plants
Source: PLoS One. 2013 Jan 25;8(1):e54836. doi: 10.1371/journal.pone.0054836 (PMC3555983; doi:10.1371/journal.pone.0054836)

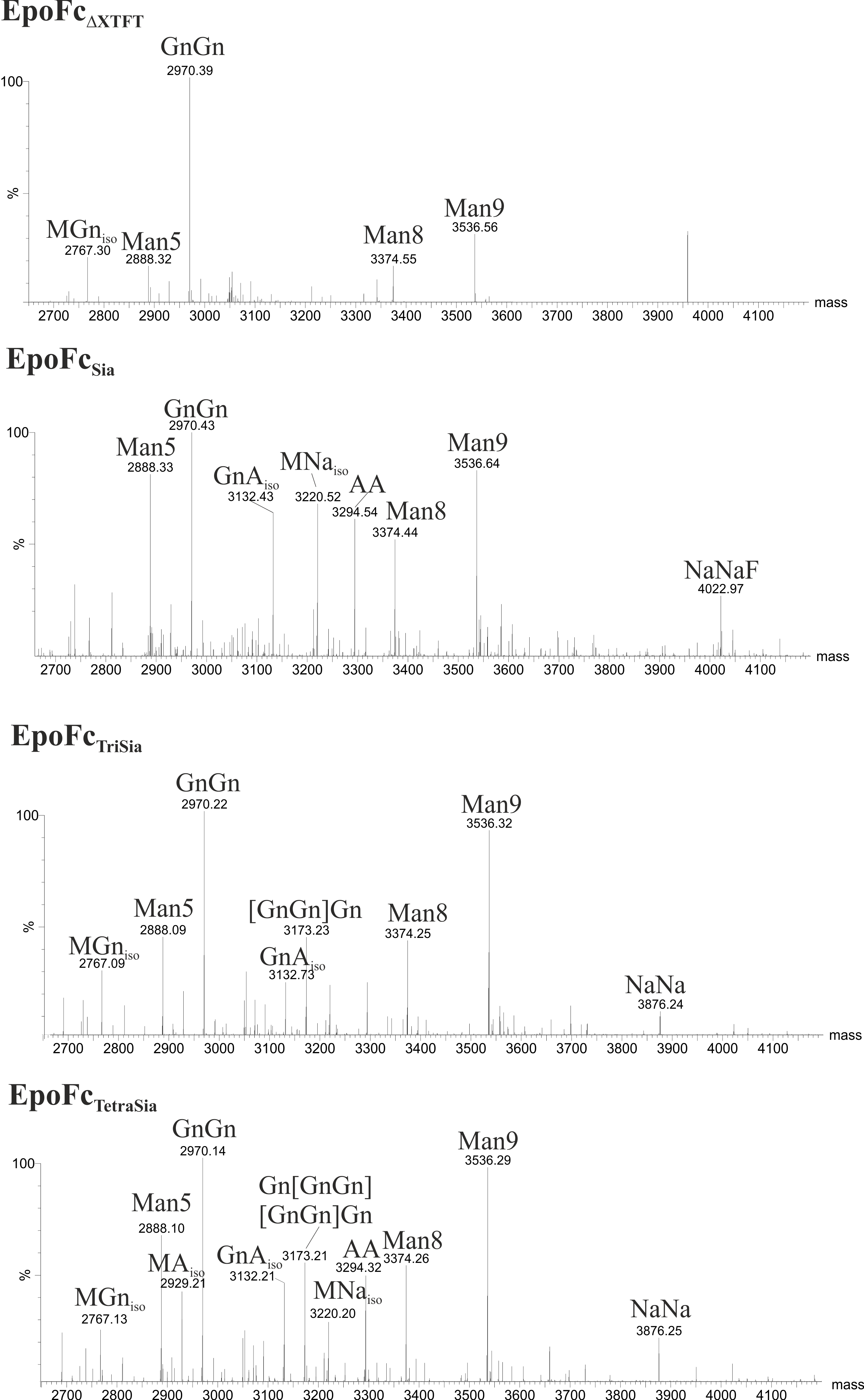

Supplement: Figure S1 — N- glycosylation profile observed in the Fc glycopeptide (R/EEQYNSTYR) of rhEPOFcΔXTFT: rhEPOFc expressed in N. benthamiana ΔXTFT mutants; rhEPOFcSia: rhEPOFc co-expressed in ΔXTFT with mammalian genes for protein sialylation; rhEPOTriSia: rhEPOFc co-expressed in ΔXTFT with mammalian genes for synthesis of tri-antennary sialylated N- glycans; rhEPOTetraSia: rhEPOFc co-expressed in ΔXTFT with mammalian genes for synthesis of tetra-sialylated N- glycans. For interpretation of glycoforms present in assigned peaks see Figure S5. (TIF) [file pone.0054836.s001.tif]

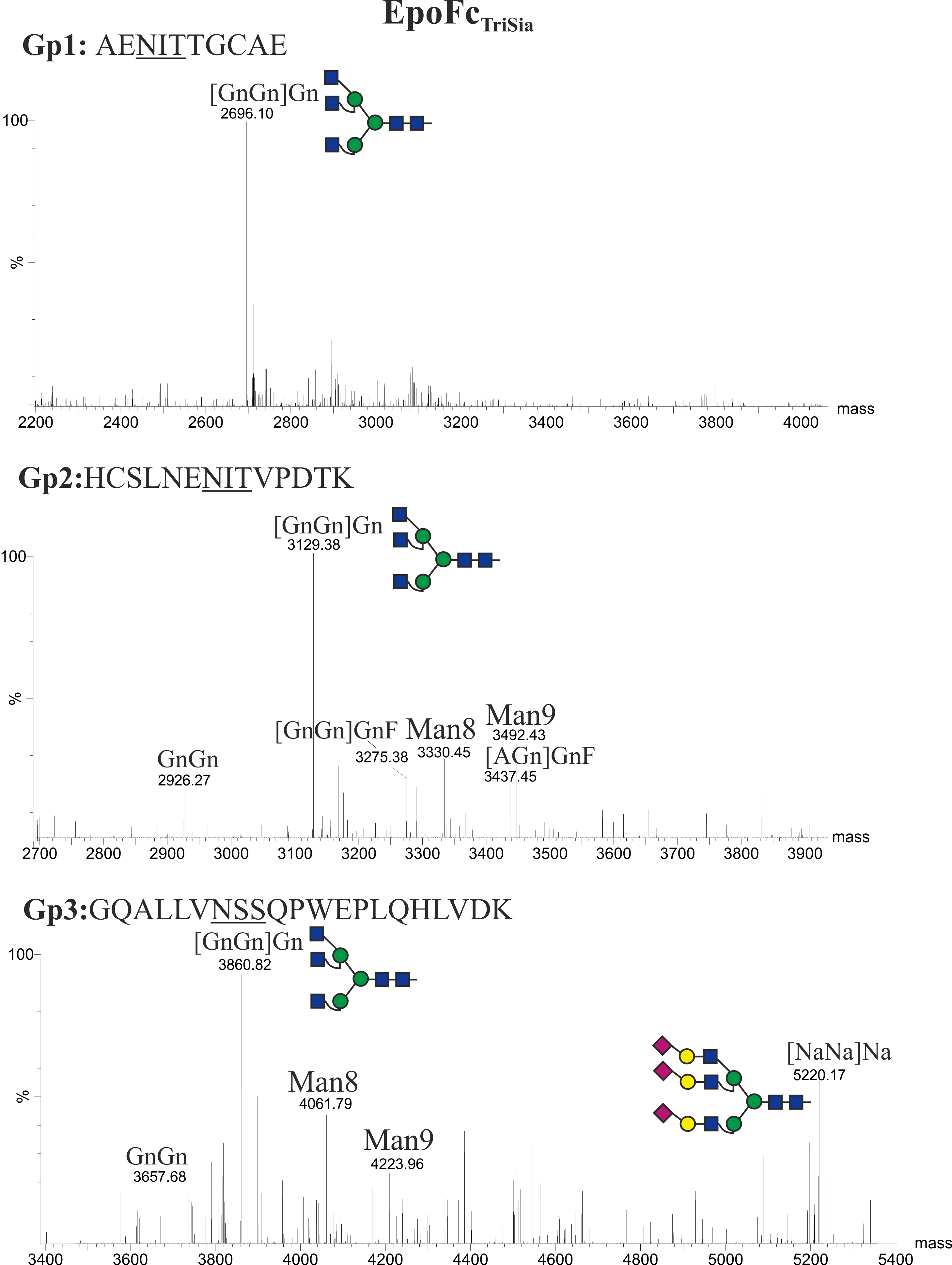

Supplement: Figure S2 — N -glycosylation profile of rhEPOFcTriSia present in fraction B of the 55kDa band ( Figure 2B , lane 3). Glycosylation patterns of rhEPO Gp1: E/A22ENITTGCAE31; Gp2: E/H32CSLNENITVPDTK45 and Gp3: R/G77QALLVNSSQPWEPLQHLVDK97 are shown. Peak labels were made according to the ProGlycAn system (www.proglycan.com). For interpretation of glycoforms present in assigned peaks see Figure S5. (TIF) [file pone.0054836.s002.tif]

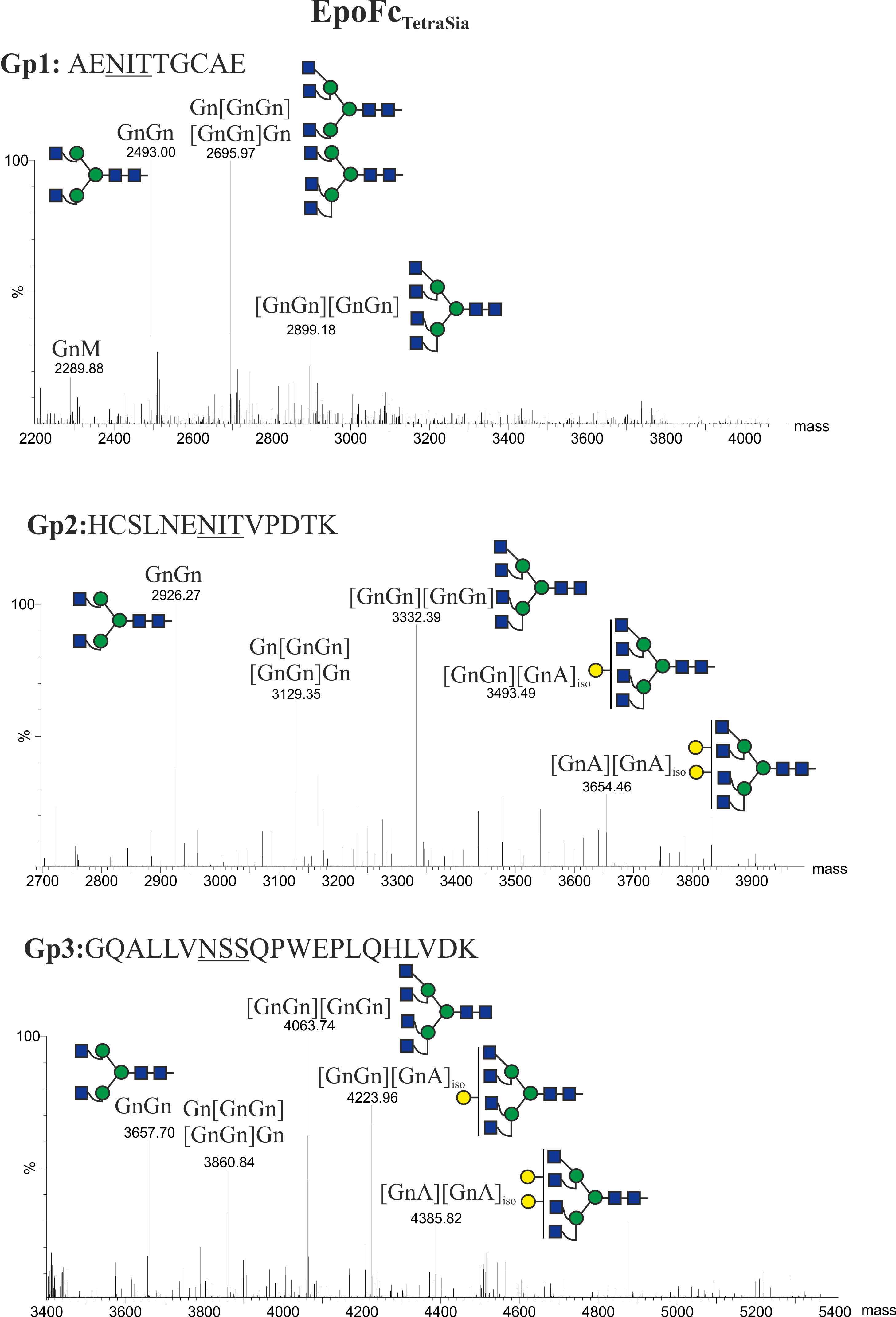

Supplement: Figure S3 — N -glycosylation profile of rhEPOFcTetraSia present in fraction B of the 55kDa band ( Figure 2B , lane 4). Glycosylation patterns of rhEPO Gp1: E/A22ENITTGCAE31; Gp2: E/H32CSLNENITVPDTK45 and Gp3: R/G77QALLVNSSQPWEPLQHLVDK97 are shown. Peak labels were made according to the ProGlycAn system (www.proglycan.com). For interpretation of glycoforms present in assigned peaks see Figure S5. (TIF) [file pone.0054836.s003.tif]

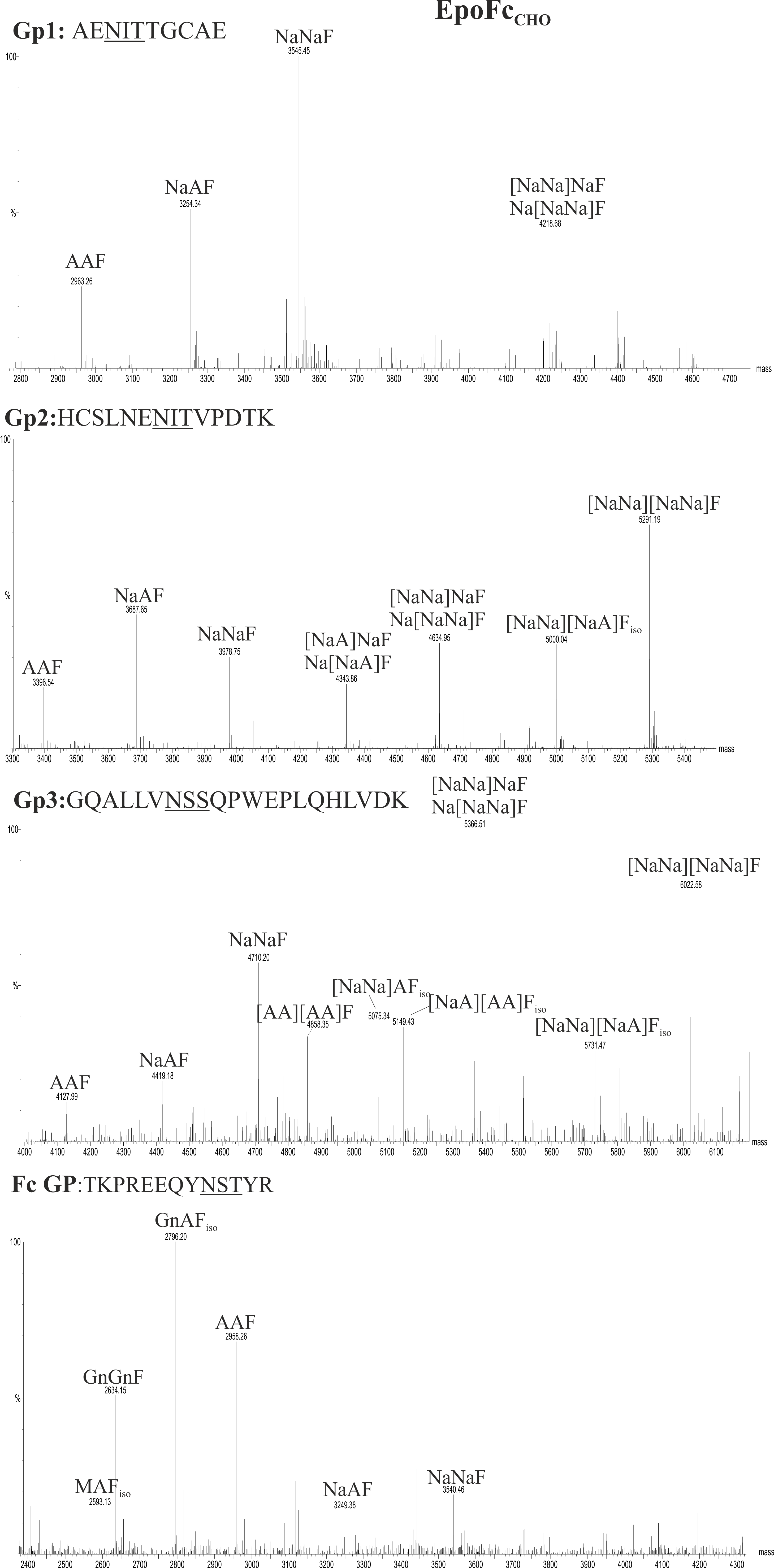

Supplement: Figure S4 — N -glycosylation profile of rhEPOFc expressed in CHO cells. Glycosylation patterns of rhEPO Gp1: E/A22ENITTGCAE31; Gp2: E/H32CSLNENITVPDTK45; Gp3: R/G77QALLVNSSQPWEPLQHLVDK97and the Fc glycopeptide (R/EEQYNSTYR) are shown. Peak labels were made according to the ProGlycAn system (www.proglycan.com). For interpretation of glycoforms present in assigned peaks see Figure S5. (TIF) [file pone.0054836.s004.tif]

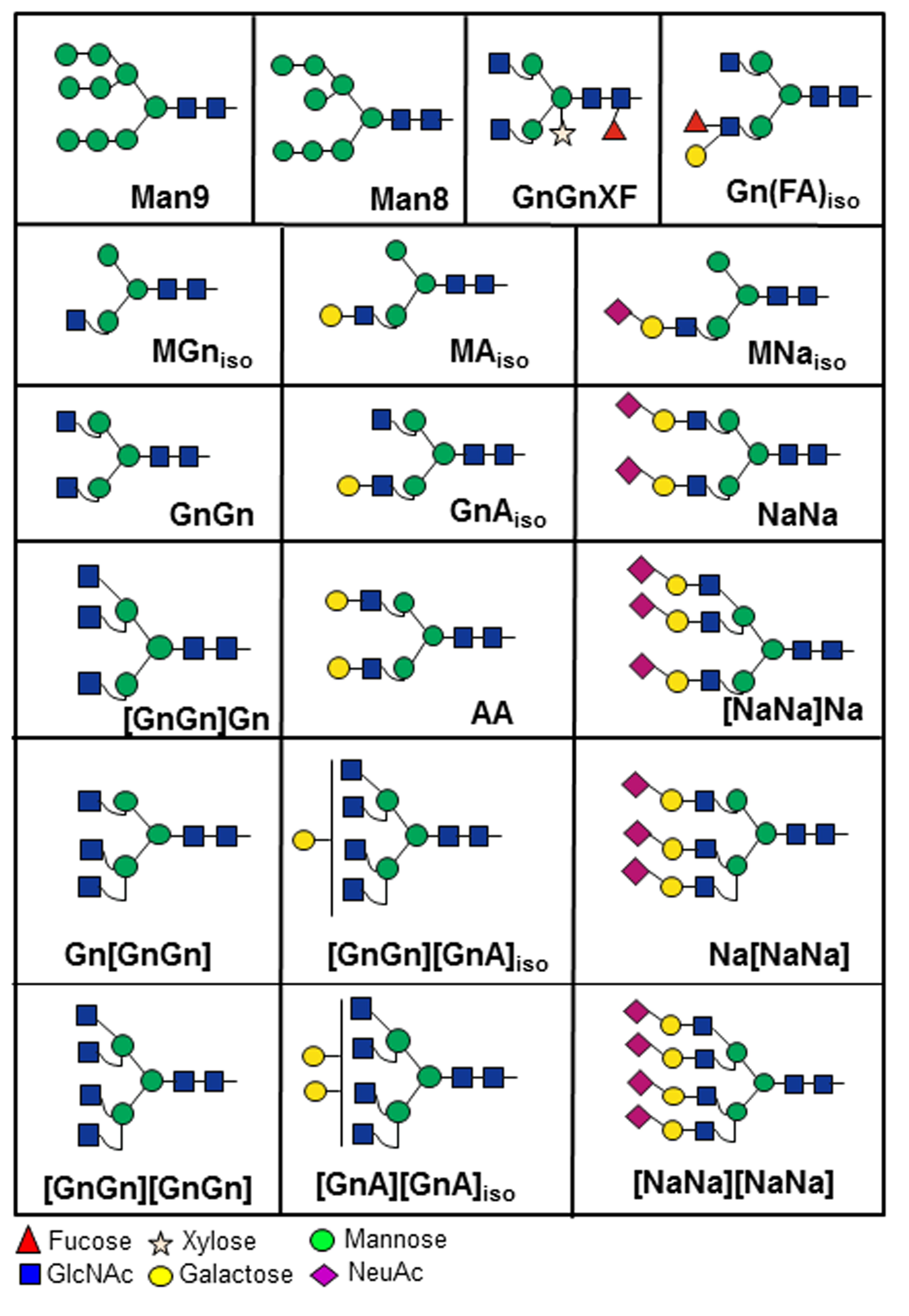

Supplement: Figure S5 — Illustration of N -glycan structures on transiently expressed rhEPOFc. Oligomannosidic structures (Man5–Man9), complex N-glycans typical of plant-derived proteins (GnGnXF) and glycans carrying Lewis-a epitopes (Gn(FA)iso) are also illustrated. Schematic representations are based on the nomenclature proposed by the consortium for Functional Glycomics. (TIF) [file pone.0054836.s005.tif]
