# Supplementary material for: Generation of Biologically Active Multi-Sialylated Recombinant Human EPOFc in Plants
Source: PLoS One. 2013 Jan 25;8(1):e54836. doi: 10.1371/journal.pone.0054836 (PMC3555983; doi:10.1371/journal.pone.0054836)
Supplement: Table S1 — List of primers as cited in Material and Methods. (DOCX) [file pone.0054836.s006.docx]

**Table S1:** List of primers as cited in Experimental Procedures

----------------------------------------------------------------------------------

**Primer Restriction Sequence (5`-3`)**

-----------------------------------------------------------------------------------

GNE F1 *XhoI* tataCTCGAGatggagaagaacgggaacaac

GNE R1 *Bgl/*II tataagatctctagtggatcctgcgcgttgtgta

NANS F1 *Xho*I tataCTCGAGatgccgctggagctggagctg

NANS R1 *BamH*I tataGGATCCttaagacttgatttttttgccatg

CMAS F1 *Xho*I tataCTCGAGatgaagcccccgcacctggcagcccta

CMAS R1 *BamH*I tataGGATCCctatttttggcatgaattattaac

CST F1 *Xho*I tataCTCGAGatggctccggcgagagaaaatg

CST R1 *BamH*I tataGGATCCtcacacaccaatgattctctc

STGalT R1 *BamH*I tataGGATCCctagctcggtgtcccgatgtcc

ST F1 *Xho*I tataCTCGAGatgattcataccaacttgaag

ST R1 *BamH*I tataGGATCCtcaacaacgaatgttccgg

----------------------------------------------------------------------------------
